# Tooth Ache Remedy

**Published:** 1877-02

**Authors:** 


### TOOTHACHE REMEDY.

Mr. C. A. Guild writes to *The Clinic* as follows: "I have found that collodion, mixed with enough carbolic acid to form a jelly-like mass (about equal parts are necessary), an excellent remedy for toothache. Take it on the end of a pine stick and place it in the cavity of the tooth. The pain will be relieved almost instantly if it depends on an exposed nerve. It is the most reliable and convenient remedy I ever tried."

